# An Artificial Intelligence-Based Full-Process Solution for Radiotherapy: A Proof of Concept Study on Rectal Cancer

**DOI:** 10.3389/fonc.2020.616721

**Published:** 2021-02-03

**Authors:** Xiang Xia, Jiazhou Wang, Yujiao Li, Jiayuan Peng, Jiawei Fan, Jing Zhang, Juefeng Wan, Yingtao Fang, Zhen Zhang, Weigang Hu

**Affiliations:** ^1^ Department of Radiation Oncology, Fudan University Shanghai Cancer Center, Shanghai, China; ^2^ Department of Oncology, Shanghai Medical College, Fudan University, Shanghai, China

**Keywords:** full-process solution, AI, rectal cancer, radiotherapy, automatic planning

## Abstract

**Background and Purpose:**

To develop an artificial intelligence-based full-process solution for rectal cancer radiotherapy.

**Materials and Methods:**

A full-process solution that integrates autosegmentation and automatic treatment planning was developed under a single deep-learning framework. A convolutional neural network (CNN) was used to generate segmentations of the target and the organs at risk (OAR) as well as dose distribution. A script in Pinnacle that simulates the treatment planning process was used to execute plan optimization. A total of 172 rectal cancer patients were used for model training, and 18 patients were used for model validation. Another 40 rectal cancer patients were used for an end-to-end evaluation for both autosegmentation and treatment planning. The PTV and OAR segmentation was compared with manual segmentation. The planning results was evaluated by both objective and subjective assessment.

**Results:**

The total time for full-process planning without contour modification was 7 min, and an additional 15 min may require for contour modification and re-optimization. The PTV DICE similarity coefficient was greater than 0.85 for all 40 patients in the evaluation dataset while the DICE indices of the OARs also indicated good performance. There were no significant differences between the auto plans and manual plans. The physician accepted 80% of the auto plans without any further operation.

**Conclusion:**

We developed a deep learning-based automatic solution for rectal cancer treatment that can improve the efficiency of treatment planning.

## Introduction

Intensity-modulated radiation therapy (IMRT) and volumetric modulated arc therapy (VMAT) are widely used in current radiotherapy clinics due to their ability to achieve desired target dose conformity and sufficient sparing of critical structures (1). There are two core tasks in a typical IMRT or VMAT planning process: target volume and organ at risk (OAR) segmentation and treatment planning. The segmentation task is performed by a radiation oncologist, and the treatment planning is performed by a dosimetrist or physicist. Both tasks require significant knowledge, experience, and time to achieve a clinically acceptable quality (2). Meanwhile, due to the complexity of technologies and the differences in personal preferences, abilities, and technical understanding, inter-physician and inter-physicist variations are inevitable (3).

Manual segmentation of target volumes and OARs remains a time-consuming task in radiation oncology (4–6). In addition, intra- and interobserver variability for most treatment sites in segmentation and the heterogeneity in clinical practices have hindered our ability to systematically assess the quality of radiation therapy plans and are considered major sources of uncertainty (4). Recently, the emerging technology of deep learning has also provided the ability to identify and automatically segment tumors or OARs on medical images. The results from deep learning-based segmentation have been promising (6). Autosegmentation has the potential to reduce the contouring burden as well as the intra- and interobserver variability in contouring.

Similar to tumor or OAR segmentation, treatment planning is another critical task in radiotherapy. It may require considerable trial-and-error to obtain a clinically accepted plan even for an experienced medical physicist (7–9). To improve plan quality and efficiency, knowledge-based planning (KBP) and script-based automatic planning have been developed (7, 10). Recently, many studies have reported that deep learning-based automatic treatment planning may be promising (9, 11).

In addition to the above two core tasks, another high time-cost task is communication between oncologists and physicists. However, both groups of healthcare professionals may have a heavy clinical workload. For large cancer centers or multi-campus hospitals, physics departments and radiation oncologists’ working areas may be physically far apart. Furthermore, as the scale of radiotherapy departments continue to expand, the time and costs of communication will inevitably increase rapidly (12).

Since artificial intelligence and machine learning look promising for radiotherapy, we seek to integrate all these technologies to minimize unnecessary operations and explore the changes that this technology brings to radiotherapy workflows. In this study, we propose a fully automatic solution for rectal cancer IMRT treatment planning. It uses a deep-learning method to accomplish contour segmentation and dose distribution prediction. By integrating with the treatment planning system (TPS) script, the workflow of radiotherapy treatment planning can be optimized. The effect of the solution will also be evaluated by an end-to-end test.

## Materials and Methods

**Figure 1:**
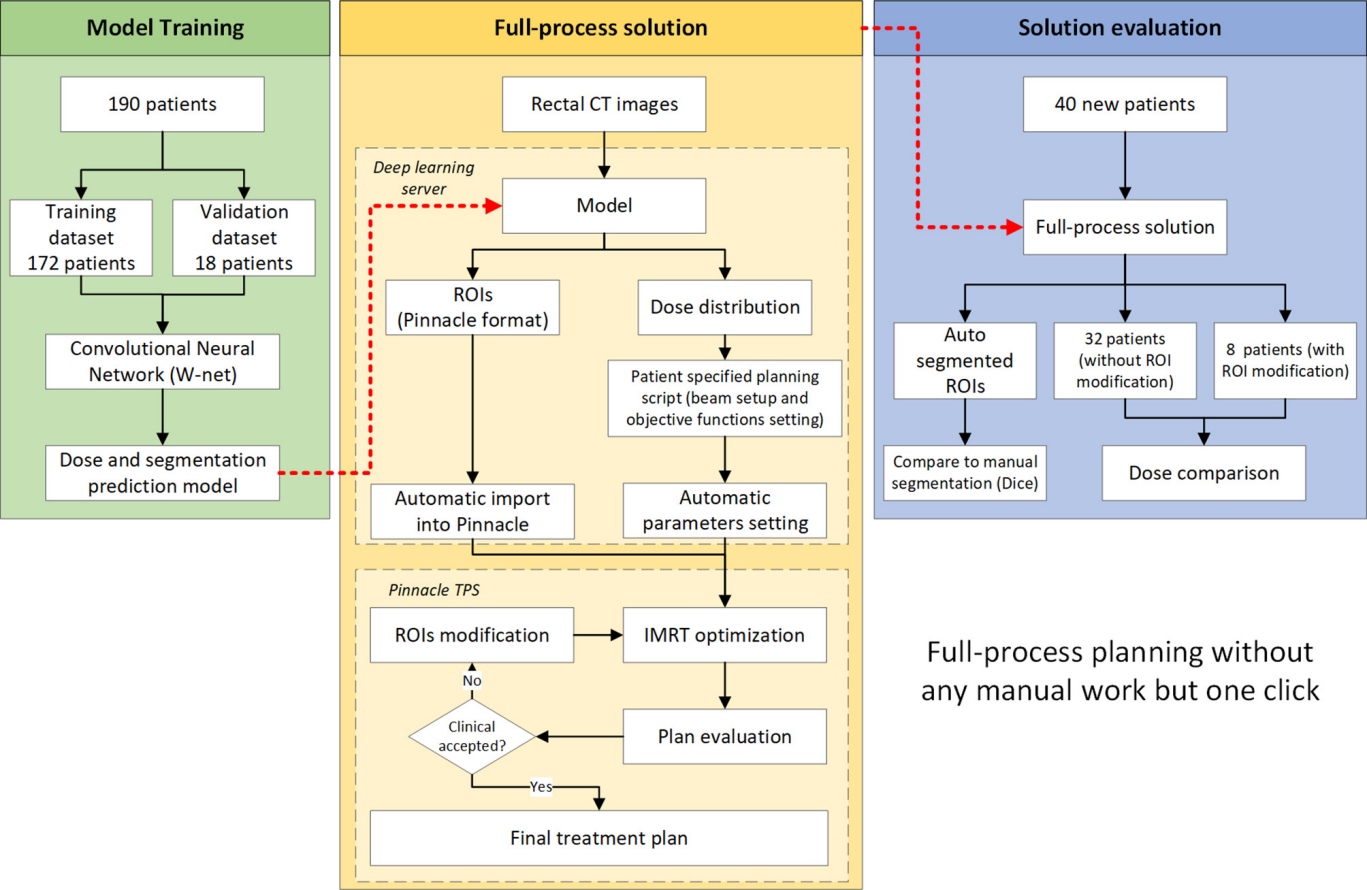
Whole automatic treatment planning process.

This study consists of three parts as illustrated in **Figure 1**. The first part consists of model training. The second part demonstrates the full-process solution workflow regarding how to use this solution in routine clinical practices. The third part is an end-to-end evaluation of our full-process solution.

### Patients and Dataset

A total of 230 neoadjuvant rectal cancer patients from 2016 to 2018 in our institution are enrolled. All patients’ tumor site was low-position (when tumor bottom is less than 10 cm away from anus). These patients were randomly selected from our clinical database with criteria of T3-4N+ staging. The planning CT was scanned one hour after bladder evacuation with the patient in the head-first supine (HFS) position. The detailed characteristics of the training, validation, and evaluation groups are presented in **Table 1**. In this concept study, the evaluation set consisted of only IMRT patients.

**Table 1:** Detailed characteristics of the patients and datasets.

	Training set	Validation set	Evaluation set
Number of patients	172	18	40
Image slice amount	10,276	1,039	2,156
PTV volume		1,041.3 ± 144.3 cm^3^	
Left femoral head Volume		76.90 ± 35.74 cm^3^	
Right femoral head Volume		77.55 ± 34.53 cm^3^	
Bladder volume		142.30 ± 85.02 cm^3^	
Small bowel Volume		775.83 ± 167.23 cm^3^	
Colon volume		110.18 ± 57.39 cm^3^	
Slice thickness		5 mm	
Reconstruct pixel size		0.9~1.3 mm	
IMRT/VMAT		149/41	40/0

### OAR and Target Segmentation

ROIs were segmented by radiation oncologists based on CT images. The left femoral head (LFH), right femoral head (RFH), bladder and clinical target volume (CTV) of the tumor were segmented. The planning target volume (PTV) consisted of a 1 cm 3D extension in all directions from the CTV. Oncologists may modify the PTV basing on their personal experience. Although small bowel and colon are very important normal organs (GI002, RTOG 0822) (13), they are not segmented in our routine clinic. For the purpose of evaluation, small bowel and colon are segmented with intelligent segmentation in uRT-TPOIS (R001.2.0.85208, United Imaging, Shanghai).

### Full-Process Solution

A W-shaped net architecture based on our previous studies (9, 14) was used in this study, consisting of a combination of two U-net convolutional neural networks (CNN). To seamlessly integrate our clinical workflow, a Pinnacle^3^-based script was developed. The entire process started after the planning CT was imported into Pinnacle, and was triggered by radiation oncologist clicking a single script button. The CT image was sent to a deep learning server in the Pinnacle image format (.img file). The server then automatically ran a deep-learning model to predict the patient’s ROIs and dose distribution. Segmentation was saved in Pinnacle ROI format (.roi file) and sent back to Pinnacle automatically. At the same time, a patient-specific sub-script was generated. DVH index are calculated with the predicted dose distribution and are used for objective functions. This sub-script was also sent back to Pinnacle, and plan generation and optimization were performed. This sub-script automatically set the isocenter, beam angle, and optimization parameters (including all objective functions and IMRT hyperparameter settings) and started the optimization. No human intervention was required during the above processes.

All patients were prescribed 5,000 cGy in 25 fractions, and the prescription definition was 97% of the mean dose of the PTV based on our clinical routine. In this study, we only used the IMRT technique for evaluation, which could be easily changed to VMAT by changing the beam type. The isocenter was placed in the geometric center of the PTV. A photon beam with 6 MV energy is used. Seven beams were used in this study with a fixed angle (180, 230, 280, 320, 40, 80, 130 degrees) with direct machine parameter optimization (DMPO). The maximum number of segments was 50, while the minimum segment area was 10 cm^2^ and the minimum number of segment MUs was 6. A maximum of 50 iterations were used for optimization. PTV was chosen as the target with a dose of 5,000 cGy.

After the optimization and final dose calculation, the plan could be immediately reviewed by radiation oncologist. If the segmentation was not clinically accepted, the oncologist could modify the contour segmentation and reoptimize the plan with another click on a script button. The reoptimization process did not change the objective function.

#### Plan Optimization

We used the Pinnacle Auto-planning module to run plan optimization. The OAR optimization goals all used max DVH, and the priority was set as low. The dose objective functions were directly calculated from the predicted dose distribution. **Table 2** shows detailed index for dose optimization. Normally, a maximum dose of 102% of the prescription and a minimum dose of 101% of the prescription dose were used for the target according to default setting of Pinnacle Auto-planning module.

**Table 2:** Optimization goals chosen in this study.

OARs	Type	Optimization goal
PTV	Max/Min Dose	102/101% prescription dose
Left femoral head (LFH)	Max DVH	V_15Gy_, V_20Gy_, V_25Gy_
Right femoral head (RFH)	Max DVH	V_15Gy_, V_20Gy_, V_25Gy_
Bladder	Max DVH	V_25Gy_, V_30Gy_, V_35Gy_, V_40Gy_, V_45Gy_

V_15Gy_ means the volume percentage of the OAR covered by an isodose line of 15 Gy.

#### Solution Performance Evaluation

Forty patients were enrolled for solution performance evaluation. The evaluation included two parts, physician assessment and objective criterion assessment.

For physician assessment, a radiation oncologist with 5 years of experience could carry out the whole process without the physicists’ help. Oncologist would assess the results of the autosegmentation and treatment planning sequentially, and were required to decide whether this plan (the Auto-plan) was suitable for delivery in the clinic. If not, the oncologist was required to provide opinions about the segmentation and plan. After that, the oncologist can modify the ROIs and trigger the reoptimization process by clicking another script button. Then, the radiation oncologist was required to reassess the plan (the Re-opt-plan). A senior oncologist who has been working for 32 years blindly reviewed all evaluation patient sets.

For objective criterion evaluation, segmentation was assessed by the dice similarity coefficient (DSC). The Auto-plan was assessed by dose-volume indices. As a retrospect study, manual segmentation and manual plans already exist. For the PTV, the max dose (D_0.01_), relative volume covered by 95% of the prescription dose (V_95_), relative volume covered by 99% of the prescription dose (V_99_), conformity index (CI), and homogeneity index (HI) were compared. In this study, HI and CI calculation were performed with the built-in algorithms of MIM (MIM Software Inc. Cleveland, OH, USA). The CI equals the ratio of the volume of the prescription dose line to that of the target, while the HI equals the ratio of the max and min doses for the target. For the femoral heads, D_2_, V_25Gy_, and V_40Gy_ were compared, while V_25Gy_, V_35Gy_, and V_45Gy_ were used for the bladder (15). For small bowel, V_35Gy_, V_40Gy_, V_45Gy_, and D_2_ were used while V_50Gy_ and D_2_ are evaluated for colon (13). We used the manually segmented ROIs as the reference set for DVH calculation since manually segmented ROIs should be considered as the gold-standard in this study.

Paired sample *t* tests were used to evaluate the statistical significance of all the dose-volume parameters between the Auto-plan and the manually optimized plan (MO-plan), as well as between the Re-opt-plan and the MO-plan. To demonstrate the final performance of our solution, we combined patients without reoptimization and those with reoptimization into the solution. A *p-value* less than 0.05 was considered statistically significant.

## Results

### Full-Process Solution

With the physician assessment, among all 40 patients who underwent solution performance evaluation, 32 (80%) patients’ Auto-plans were clinically accepted without contour modification and reoptimization. Eight patients’ plans needed target (PTV) modification. After modification and reoptmization, evaluation set plans were reviewed by senior oncologist all became clinical acceptable. The whole process would take 7 min without contour modification for one patient. Contour modification cost average 13.3 min, ranging between 6 and 20 min. Reoptimizing process cost 5 min. In comparison, manual segmentation would take more than 30 min and manual plan would take 30–40 min.

### Autosegmentation Assessment

**Figure 2:**
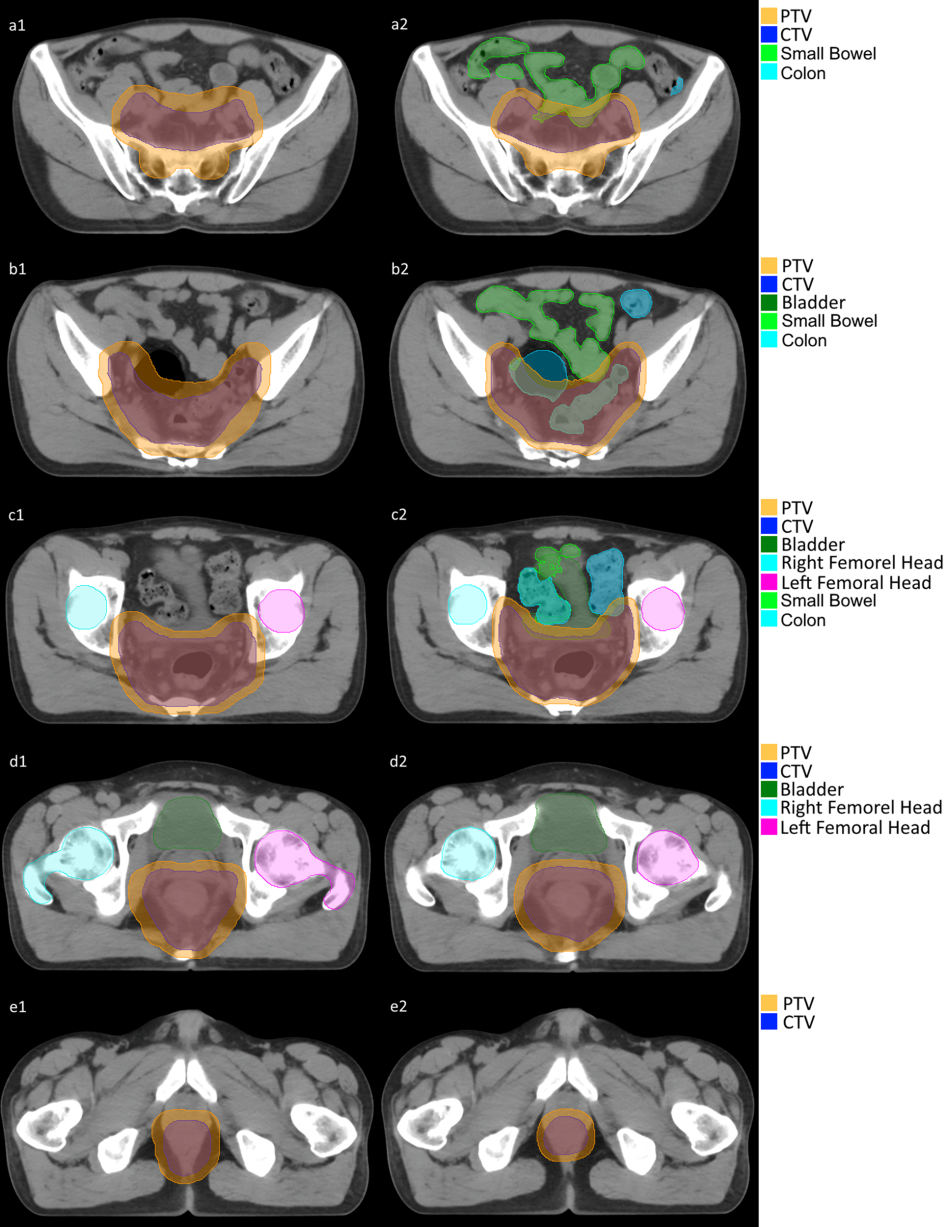
Segmentation comparison between manual (a1-e1) and auto (a2-e2).

The DSC between predicted and manual PTV segmentation was greater than 0.85 for all 40 patients (16). The mean DSC of the OARs was also greater than 0.75 but with some deviation. Here is detailed statistic (mean ± standard deviation): PTV 0.90 ± 0.02; CTV 0.87 ± 0.06; Bladder 0.80 ± 0.11; LFH 0.79 ± 0.10; RFH 0.80 ± 0.10. **Figure 2** is demonstration of a certain patient’s segmentation comparison between manual segmentation and autosegmentation.

### Auto-Planning Assessment

**Figure 3:**
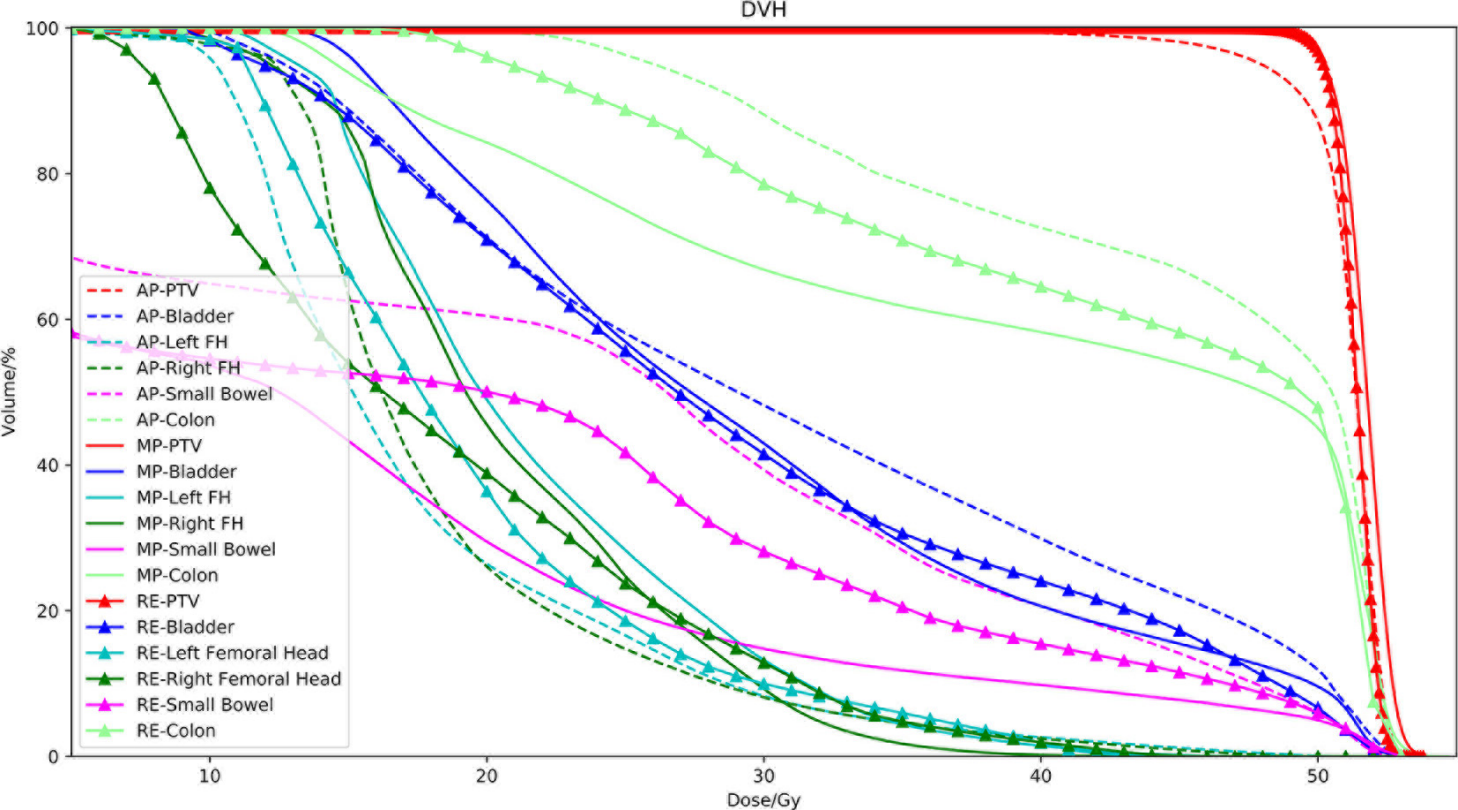
DVH curve of the MO plan, Auto plan and Re-opt plan.

The dose-volume indices are presented in **Table 3**. There were no significant differences between the Auto-plan and the MO-plan for any of the OAR dose-volume indices except femoral head V_25Gy_, which was reduced by auto-planning. Volume dose for Auto-plan and Re-opt-plan of both small bowel and colon are higher than manual plan (exceeding IMRT planning constraints, but still under compliance criteria). Although there were no significant differences between auto-plan and manual plan for max dose. The PTV coverage was significantly lower for auto-planning without reoptimization. After contour modification and reoptimization, the PTV coverage was improved; the V_99_ was better than that of the manual plans, and the CI improved from 1.12 to 1.09. An example set of DVH curves was shown in **Figure 3**. This patient’s autogenerated plan was considered clinically unacceptable by our physician. After contour revision and plan reoptimization, the plan was considered acceptable.

**Table 3:** Statistical comparison among the MO-plans, Auto-plans, and Re-opt-plans.

	MO-plan ± SD	Auto-plan ± SD	Auto *vs.* MOP value	Re-opt-plan ± SD^#^	Re-opt *vs.* MOP value^#^
PTV D_0.01_, Gy	53.6 ± 0.3	53.8 ± 0.24	.029^*^	53.7 ± 0.2	.051
PTV V_95_, %	99.93 ± 0.10	97.94 ± 1.53	<.001^*^	99.97 ± 0.02	.076
PTV V_99_, %	98.64 ± 0.79	95.29 ± 2.33	<.001^*^	99.09 ± 0.24	.012^*^
PTV HI,	1.08 ± 0.01	1.09 ± 0.01	.006^*^	1.09 ± 0.01	.005^*^
PTV CI,	1.04 ± 0.03	1.12 ± 0.09	.002*	1.09 ± 0.03	<.001^*^
Bladder V_25Gy_, %	66.90 ± 17.12	70.35 ± 9.48	.316	67.21 ± 12.96	.899
Bladder V_35Gy_, %	41.79 ± 15.06	46.90 ± 11.58	.055	42.69 ± 12.00	.616
Bladder V_45Gy_, %	21.77 ± 8.54	24.70 ± 8.81	.091	20.77 ± 7.63	.269
Left femoral head D_2_, Gy	40.40 ± 4.26	39.71 ± 4.51	.417	40.44 ± 4.65	.948
Left femoral head V_25Gy_, %	36.46 ± 14.15	19.15 ± 9.32	<.001^*^	20.00 ± 6.93	<.001^*^
Left femoral head V_40Gy_, %	2.66 ± 2.90	2.60 ± 2.81	.924	2.80 ± 2.19	.759
Right femoral head D_2_, Gy	39.00 ± 4.60	40.83 ± 5.45	.076	39.43 ± 4.50	.543
Right femoral head V_25Gy_, %	33.33 ± 11.57	19.20 ± 8.11	<.001^*^	20.55 ± 6.04	<.001^*^
Right femoral head V_40Gy_, %	2.11 ± 2.90	2.95 ± 2.26	.037^*^	2.99 ± 2.09	.054
Small bowel V_35Gy_, cm^3^	105.76 ± 75.37	150.68 ± 87.40	<.001^*^	139.76 ± 88.13	<.001^*^
Small bowel V_40Gy_, cm^3^	84.83 ± 65.14	114.75 ± 76.42	<.001^*^	104.16 ± 75.04	<.001^*^
Small bowel V_45Gy_, cm^3^	67.29 ± 56.01	86.41 ± 62.71	<.001^*^	77.19 ± 60.50	<.001^*^
Small bowel D_2_, Gy	49.29 ± 5.82	50.4 ± 3.26	0.088	49.77 ± 4.97	0.025^*^
Colon V_50Gy_, %	48.67 ± 25.34	54.26 ± 24.01	0.033^*^	50.93 ± 24.90	0.039^*^
Colon D_2_, Gy	52.45 ± 0.31	52.59 ± 0.24	0.068	52.43 ± 0.30	0.898

The mean value and standard deviation are shown.

^#^Reoptimization was performed for eight patients. The results presented in this column are from all 40 patients’ data. These results can be considered the “final results” of the solution.

^*^Statistically significant.

## Discussion

In this study, we developed an integrated solution for radiation segmentation and planning. This solution can significantly increase the efficiency of the whole workflow of radiotherapy. Two core radiotherapy tasks were integrated into one task with artificial intelligence. Since high-quality plans can be directly generated for 80% of rectal cancer patients by a physician alone, the communication cost could be largely reduced (17). Although 20% of the automatically generated plans were clinically unacceptable, with a reoptimization step, these plans become clinically acceptable.

In the oncologist’s evaluation, the quality of 80% of patients’ plans directly satisfied the clinical criteria following automatic segmentation and automatic planning. Twenty percent of the patients required contour modification and reoptimization. After reoptimization, all plans satisfied the clinical criteria. Dose differences between auto-plan and re-opt-plan are mainly arose by segmentation since we didn’t change any other parameters. The dose-volume index comparison also demonstrated that after reoptimization, the differences between the Auto-plans and the MO-plans were reduced.

The radiotherapy processes for rectal cancer in our institution, including segmentation and treatment planning, are consistent. All radiation oncologist and physicists followed one unified consensus in segmentation and treatment planning. This consensus was “learned” by the deep-learning neural network. It’s difficult to extend the entire scheme to multiple centers since enormous differences exist in segmentation and plan, especially when radiation oncologists have their own unique experiences besides contouring atlases. Though recent study has been focused on model adaption, lacking a unified guide is still a major hindrance to extend deep learning’s application (18).

There were differences in the HI and CI of the PTV and the indices of the femoral heads, however. We believe this was mainly caused by the Pinnacle Auto-planning module, which relaxes the PTV constraint to reduce some OAR doses. Although obtained a high DSC value for the PTV (>0.85), the system did not perform well in high-risk lymph node drainage areas and recrudescence areas. For example, the sacral, inferior mesenteric, internal lilac, external lilac, and inguinal lymph nodes are all considered high-risk clinical target volumes. We observed that these parts were frequently modified by physicians during the evaluation process. This indicated that a physician’s assessment and evaluation was still required. In clinical use, oncologist could modify segmentation of PTV while auto-planning is ongoing. If the segmentation doesn’t need modification, the plan can be evaluated immediately.

We evaluated radiation dose for small bowel and colon in this study. Although Auto-plan and Re-opt-plan dose for small bowel and colon are higher than MO-plan, they still met RTOG 0822 compliance criteria. Higher dose for small bowel and colon may be caused by lower dose for femoral heads. Since dose deposition along 90/270 degree decreased, dose deposition along 0/180 degree increased where small bowel and colon are located. Besides according to a phase 3 clinical trial in our center (19), the incidence of grade 3 or higher diarrhea was only 3.5% when combining pelvic irradiation concurrently with 5-Fu monotherapy.

This study combined knowledge-based and script-based planning. Script-based automatic approaches have been proven to have great potential for reducing workloads in clinical radiotherapy (20, 21). We use the Pinnacle Auto-planning module only because it is clinically implemented in our institution (22). Knowledge-based planning has been used to aid in the design of radiation treatment plans for decades (23). The main advantage of KBP is that it can provide an achievable goal before optimization. This goal may also reflect the physician’s tradeoff between target and OAR dose coverage. Here, we used deep learning to generate the dose distributions and transfer them to the TPS as DVH constraints. This is because at present we do not have an optimization engine that can directly incorporate dose distributions as its goal.

There were some limitations in this proof of concept study. First, the plan chosen for training might not be the optimal plan. As we have seen, with a slight compromise in PTV dose heterogeneity, our solution reduces the patient’s femoral head dose. This indicates that some routine clinical plans may not focus on reducing the femoral head dose. Meanwhile, although we predicted the dose distribution in every voxel, we do not use it directly in the optimization step. Because current commercial TPSs only accept DVH indices as optimize objectives, this can be improved in the future.

We chose rectal cancer to demonstrate our approach because it is simpler than cancers at other sites. These complex sites (such as the lung) have complex target segmentation and beam angle selection protocols. As it stands, our solution is not suitable for these sites. This requires further investigation.

## Conclusion

The full-process solution developed in this study integrated automatic segmentation and treatment planning. This solution can significantly increase the efficiency of the whole workflow of radiotherapy.

## Data Availability Statement

The original contributions presented in the study are included in the article/supplementary material. Further inquiries can be directed to the corresponding authors.

## Ethics Statement

The studies involving human participants were reviewed and approved by Fudan University Shanghai Cancer Center Institutional Review Board. Written informed consent for participation was not required for this study in accordance with the national legislation and the institutional requirements.

## Author Contributions

XX: paper writing. JZW: network framing. JP, JF: model training. YL, JZ, JFW, ZZ: segmentation and plan evaluation. YF: data collection. All authors contributed to the article and approved the submitted version.

## Funding

This work is supported by the National Natural Science Foundation of China (No. 11675042, No. 11805039, No. 11805038) and the Imaging Funding of Fudan University Shanghai Cancer Center (YX201703) and the Shanghai Committee of Science and Technology Fund (19DZ1930902) and Xuhui District Artificial Intelligence Medical Hospital Cooperation Project (2020-009) and the Varian Research Grant (The deep learning based 3D dose prediction and automatic treatment planning).

## Conflict of Interest

The authors declare that the research was conducted in the absence of any commercial or financial relationships that could be construed as a potential conflict of interest.
